# Contributions of ambient assisted living for health and quality of life in the elderly and care services - a qualitative analysis from the experts’ perspective of care service professionals

**DOI:** 10.1186/1471-2318-14-112

**Published:** 2014-10-18

**Authors:** Christian Siegel, Andreas Hochgatterer, Thomas Ernst Dorner

**Affiliations:** Centre for Public Health, Institute of Social Medicine, Medical University of Vienna, Kinderspitalgasse 15/1, Vienna, 1090 Austria; Biomedical Systems Division, Department of Health and Environment, AIT Austrian Institute of Technology, Viktor-Kaplan-Strasse 2/1, Wiener Neustadt, 2700 Austria

**Keywords:** Ambient assisted living, Quality of life, Independent living, Independent elderly, Assistive technologies

## Abstract

**Background:**

Because of the demographic change in industrial countries new technical solutions for the independent living of elderly will become important in the next years. Ambient Assisted Living seeks to address the upcoming challenges by providing technical aids for elderly and care givers. Therefore it is crucial to understand how those socio-technical solutions can address their needs and quality of life (QOL). The aim of this study was to analyse the main needs of dependent elderly and to investigate how different solutions can contribute to health and quality of life.

**Methods:**

A qualitative study design consisting of interviews with 11 professionals of geriatric care organisations was chosen. The data analysis was done by applying the qualitative content analysis by Philipp Mayring. The analysis was based on the basic principle of the bio-psycho-social model of health

**Results:**

Ambient Assisted Living solutions and assistive technologies can have positive impacts on different dimensions of health and quality of life. The needs and problems of elderly can be addressed by applying appropriate solutions which influence the physical, mental and social dimensions of quality of life. There are also benefits for social care providers, their staff and caring relatives of impaired elderly. Ambient Assisted Living solutions can also be used as a facilitator for operational optimization of care services.

**Conclusions:**

Solutions for telemedicine and telecare which are connected to Ambient Assisted Living solutions will have the biggest positive impact on care giving services. Also simple technical aids can be beneficial for elderly to enhance QOL by enabling autonomy in their familiar surroundings.

## Background

In the following centuries the upcoming demographic change in most of the developed western countries will lead to big social and economic challenges in the daily life and care of elderly people [1]. It will become necessary to develop solutions which facilitate social support for old people, enable workforce availability and make the geriatric care of old people more cost effective for funding the healthcare systems. One of the major challenges in geriatric care is the maintenance of independency and prevention of institutionalization [2]. To address this one approach is to give elderly the possibility to live in the own home as long as possible by applying new technology-based solutions which support a widely self-determined life of aged people. The aim is to combine information and communication technologies and the social environment of elderly to develop new concepts, products and services for their daily life. In Europe such solutions are developed under the term “Ambient Assisted Living (AAL)”.

AAL solutions will become economically important for formal as well as other stakeholders involved in care [2]. This is because the mostly physically limited elderly people will get new possibilities to organize their life more independently within their familiar surroundings. By giving aged people the chance to live an extensive independent life, the need for care could be reduced.

Currently, a big number of research projects and public financed initiatives (EU-Framework 7 Programme - Projects) started in the last years (e.g. AAL Joint Programme [3], AALIANCE (1 and 2) [4], etc.). These efforts usually focus on usability aspects and the technical feasibility of products.

In most AAL projects it is assumed that the developed technologies and services will improve the quality of life (QOL) and well-being of elderly people. Unfortunately the impact on health and quality of life was documented in only few scientific publications. For example the results of a research project which focussed on the impact on health by adapting of light conditions showed that AATs can have a positive effect on health and quality of life [5]. The concept of QOL represents one of the most important outcome parameters of health promotion interventions, especially in elderly people [6], and is therefore a central point to measure if clinical as well as non-clinical interventions have positive effects on the study population [7, 8].

The aim of this study was to explore the influential aspects of AAL solutions on health and QOL of elderly people from the experts’ point of view. Their professional role in the field of care giving services gave the chance to take a look behind the marketing aspects of AAL and to investigate how these technologies can contribute to the daily life of aged individuals.

## Methods

### Design of the study

To gather appropriate information we conducted an explorative qualitative study consisting of problem- focused expert interviews. This method is an established method in qualitative research to collect specialized information and perceptions about specific circumstances from experts. By performing expert interviews, the interviewee doesn’t represent the object to be investigated, in fact he or she is the medium which transports and reports the specific knowledge about the topic which is in focus of research [9]. The perceptions of experts can differ from the accessible public information because of the particular role in an organization where the person is faced with situations which are not common for people in general public (for example:. the chief executive officer of a care giving organization can have a broader or deeper understanding of available technical solutions for care than the relatives of dependent elderly). In this study the important advantage of the qualitative approach is to have the chance to gather realistic and experience-based information which can’t be easily analysed by numerical or statistical approaches as a part of quantitative research.

### Sample

From February to June 2012, 13 experts were contacted via telephone and email to ask for participation in this study. The aim was to conduct interviews with experienced staff of care giving organizations which provide mobile care services. The experts additionally must have existing knowledge about the concept of solutions in the field of Ambient Assisted Living.

The email addresses and phone numbers have been searched on the publicly available websites of the care giving organizations. Out of the 13 contacted institutions 10 declared that they are willing to participate in this study. In one case, two representatives of one social care institution participated in the interview that lead to a total number of 11 interviewees within 10 interviews. The conversation with the interviewees lasted – based on the available time resources – for 22 to 102 minutes. Nine interviews were recorded, and afterwards transcribed almost verbatim according to a set of rules which was developed according to Kuckartz [10]. Only one expert did not want to be recorded during the interview. In this case the answers were documented in an interview-protocol.

The sample of the participants separated in gender, profession and the federal state for which they are responsible for is summarized in Table 1.

**Table 1:** **Sample of participating experts (N =11)**

Gender	
Female	n =6
Male	n =5
**Profession**	
Chief executive officer of home care institution	n =3
Head of care services (extramural facilities)	n =5
Social care worker	n =2
Head of public relations	n =1
**Federal state**	
Burgenland (country)	n =1
Lower Austria (country)	n =2
Vienna (city)	n =8

### Data collection

The problem focused expert interviews were executed in the organizations’ offices of the interviewees. The interviews were semi-structured and conducted by CS. In advance of the interviews, an interview-guide was developed, based on the following main research questions:

-  What are the most important needs and challenges for elderly?
-  How can assistive technology and Ambient Assisted Living solutions beneficially contribute to the needs and challenges of elderly subjects and care givers?

The used interview-guide also included questions which dealt with the economic impact of AAL solutions. The reported content of these questions did not relate to our research questions and is therefore not part of this publication.

Before conducting the interviews, the objectives of the study were explained to the participants and the informed consent was subscribed by the experts. During the interview-process, the interviewer explained the questions accurately to make sure that the questions were not misunderstood.

### Ethics approval

The application for the study was submitted to the ethics committee of the Medical University of Vienna. The application was approved with the vote number 1546/2012.

### Data analysis

The expert interviews were carried out between May to August 2012. One Interview was conducted with each expert. The data analysis was performed with structured tables in Microsoft Excel®, based on the approach of the qualitative content analysis of Philipp Mayring [11]. Key findings were identified, paraphrased and generalized by CS. Afterwards the generalized item was reduced to main categories and sub-categories by CS. The inductively developed categories were re-checked to the paraphrased and generalized material to prove consistency and real meaning by CS and TD. The whole analysis was based on the bio-psycho-social model of George L. Engel [12] which provides a more holistic approach of health and disease as it involves factors which influence the physiological, psychological/mental and social health.

In the course of the analysis the aspects of interpretation and the reduced items were continuously developed near to the material and the context of the described answers of the experts. To make sure the topic was sufficiently investigated, the interviewer paid attention on the saturation and comprehensiveness of the study material. After the 8^th^ interview, the saturation of the content was reached.

The cited quotations in this publication were translated from German into English. At this point it is important to notice that the language of the interviewees was coloured by different Austrian dialects and idioms. To keep the real meaning of the reported facts, the dialectic content of the transcribed interviews was only partially translated into English. The quotations were numbered with the coded interview partner and the starting line number of the citation in the transcribed material (e.g. “R3” for “respondent number 3”).

## Results

As the first step in our process of analysis we identified the needs and challenges of elderly which were reported from the experts’ perspective (main category “Individual demand”). In the second step we identified the reported basic and advanced technologies and their contributions to organizational aspects for care service provider (main category “Technology for care”). The third main category (“Health and quality of life by AAL”) summarizes the findings of the individual’s benefits of AAL solutions.

### Individual demand (C1)

This category defines two sub categories (see Table 2). It describes the most important individual needs and individual problems of elderly with demand for support by care giving organizations or their relatives.

**Table 2:** **Categorisation of the results**

Main categories	Sub categories
**C1: Individual Demand**	C1.1: Needs of elderly
	C1.2: Problems of elderly
**C2: Technology for Care**	C2.1: Basic technologies for care
	C2.2: Advanced technologies for care
**C3: Health and quality of life by AAL**	

### Needs of elderly (C1.1)

In general the experts agree on the point that dependent elderly want to be treated as competent and sovereign individuals, regardless from the disabilities they are suffering from. They have the desire to be supported in a loving way. The individual’s needs are influenced by the personal circumstances of their life and social environment:

*‘…we do have seniors who put make up on before the nurse comes to visit them. Often this is neglected.’* (R4/123)

*‘…it sounds like a buzz phrase, but it is not a natural consequence to interact at an eye level with those people – no matter how disoriented they are. It is very important to take them serious and not giving them the feeling of obtrusiveness…’* (R2/101)

The need of continuity, familiarity and respect plays a major role for the dependent persons. If changes in their surrounding are necessary these things always must be done step by step.

*‘…, I mentioned that familiarity is very important to cope with the daily life…’* (R2/122)

*‘… the biggest possible degree of continuity by considering the individual’s needs in the surrounding conditions. This could be banal things, e.g. it is very important for a person to take the shoes off before entering the flat…’* (R8/33)

*‘… my caregiver should come always at the same time, be on time, should be very friendly. (…) That’s it. The stability.’* (R9/479)

The elderly do have a strong desire for social interaction, especially with their family members. Also the fulfilments of their wishes is an important need and therefore to be considered by care giving staff and relatives. Two interviewees reported that consuming delicious food plays a major role for aged people because they love to enjoy indulgence as same as young people do. Here, talks about delicious experiences of meals are used as an instrument to create a level of joint interaction and social interactions. In the interviews, it was also reported that the experience of fun in old age is also an important need. But the fulfilment of this very important need is a rare phenomenon.

*‘…The social interaction is an important thing. Gadgets do not fully replace this but they make fun…’* (R7/705)

*‘…Food is the main topic, everything. What is the meal today and what will the meal be tomorrow and what did we have yesterday? For elderly food does play the same role as sex for pubescent…’* (R9/487)

The fulfilment of elderly’s′ individual needs like delicious food and the feeling of being secure in the familiar surroundings have positive impacts on QOL.

*‘…the process of having meal as a manner of indulgence. It should have usually play this role for people… food enables social contact, it is living…’* (R9/502)

The experts agree that improving social and personal interaction does influence quality of life and can lead to improved health status by influencing mental health. Furthermore, the communication between relatives and seniors at the one side and between care givers and elderly on the other side does have influence on the well-being of the old person.

*‘…if he goes to a day care centre, the lust for life and emotional appeal. Those minds are in good shape…’* (R7/100)

*‘…we say … the social contact is the thing that really helps …’* (R9/169)

*‘…we take care that relatives do visit the residents … that they communicate. But also when our care staff is visiting the assistive living accommodations they talk to them …’* (R10/18)

*‘…autonomous and self-determined, but they have the possibility to be a part of our big team …’ (R10/30)*

From the perspective of care staff or care giving relatives, a personal relationship and appropriate personal interaction as well as the possibility to cooperate with the dependent person is highly necessary. The necessity of personal interaction with a familiar assistant is also a main need, especially when the support is on a basic and intimate level of interaction (e.g. feeding, toilet support, personal hygiene).

*‘…as often as possible the same care person. Being on time. Appearance. Reliability. These are the most important aspects…’* (R8/464)

*‘…and the social element he does need. This is the aspect which is claimed by the most of them…’* (R7/722)

*‘..the social dimension is one of the most important ones. In other respect it is “warm, to be full, clean”. Some people are embarrassed because of incontinency. They do not want to smell bad; they do not want to lie in a wet bed. Here simply the human being is needed…’* (R7/716)

The QOL can be improved by giving dependent people the possibility to act autonomously within their familiar surroundings. Autonomy can be facilitated and improved by equipping the home with barrier-free gadgets, helping aids and technical gadgets. The main objective is to facilitate the activities of daily living in the elderly (for example: bathing, dressing, etc.).

*‘…most important are low barriers, barrier-free surroundings, I mean in the flat and outside the flat and then the near infrastructure. But it is, this is a key point, the barrier-free, to have different helping aids in the flat which facilitate a lot of things…’* (R6/88)

The need for autonomy could be addressed by (non-technical) services which provide solutions for challenging situations for impaired people. They improve autonomy by giving situational support when it is needed (e.g. cleaning services and other tasks in the household). An expert said that moving to an assisted living facility can lead to improved autonomy, because of the additional services in those homes. Nevertheless, for dependent elderly it is crucial to stay motivated and activated by their care givers to do things on their own and keep them mobile as long as possible.

*‘… it would be easier to open the oven like a wing from the side, like a door of a cupboard, and below of this I can pull out a place of deposit and take the baking tray on it…’* (R6/98)

*‘… imagine the following: … You have problems with your legs and you have to clean up the flat. How could you do that? Impossible. But you are dissatisfied, because you see that your flat is not administered enough. This means you need someone to do it. … – this assisting person visits you and does this work with you together…’* (R10/68)

An interview partner said that the autonomy of making decisions about the own body and the home surroundings is a possible aspect, how health and QOL can be improved.

*‘…or it (autonomy) is about the human being who lives here and can decide on his or her own if there is a spot of dirt or not. That is his right to say, “This spot I want to have here. And when I want to have bread crumbs on my table, I want it this way”…’* (R9/310)

### Problems of elderly (C1.2)

The most important problems in elderly are physical caused, cognitive limitations, tremor and impairments in mobility. Especially the limitations in mobility lead to declining social interactions and sometimes feeling insecure and imprisoned at the own home. One interview partner stated that losing the mobility means being imprisoned at your own home.

Also mental changes in life of elderly due to dementia are part of the most crucial problems. They lead to disorientation and can have a huge negative impact on the activities of daily living.

*‘…people are often able to walk around at one level of a building but they can’t get upstairs more than 3, 4 or 5 stairs and are therefore more or less prisoners in their own flat…’* (R8/57)

*‘…another topic is the tremor …’* (R8/64)

*‘…under some circumstances also the social surrounding is reduced because of the limitations in mobility , and furthermore the psychological changes which come along with ageing. Also in the direction of being disoriented, dementia diseases which impede activities of daily living…’* (R2/29)

*‘…and for most of the elderly the fear of intrusion of strangers, fear to be alone, fear of isolation…’* (R9/163)

Another identified problem is the thinning of the social environment because of deceased family members and friends. Furthermore, from the expert’s view, in today’s common perception of society an old person seems not as valuable as a young person. Especially aged people feel this perception which leads to a feeling of not being needed anymore and depressive mood or psychological disorders.

*‘…And then, the social losses, that people often lose their most important persons in this phase of life’ (R2/28)*

*‘…because the old person is unpresentable. In Austria, this aspect de facto does not exist (…)’ (R8/127)*

*‘…from the perspective of the old people you go to an assisted living home or a nursing home and hand over the individuality at the entry door. That’s the way it is, yes…’* (R9/284)

Another domain of problems for elderly was explained by the insufficient financial resources. This is especially a problem for old women and their relatives.

*‘…Furthermore, there are the social problems; most of them do not have a good financial background, the pensions of old women are at the bottom limit …’* (R2/25)

*‘…I do see it in my own family, we additionally paid, because it was not enough (money)… no problem for paying additional for our mother. They (the government) will not pay additional for it …’* (R7/876)

The next problem is the transition to the new situation of the need to be cared or supported in any way. For this new unfamiliar situation there are not enough institutions that have the possibility to give appropriate advice to elderly and their relatives.

*‘…there is no good advisory service for old people!… it is all about “how do I motivate people to make use of assistance at home?”. They have to go a step forward and allow an unfamiliar person to get into the flat and I have to admit that I am not able to do it anymore. This is the crux of the matters in life…’* (R7/340)

### Technology for Care (C2)

In this main category two sub categories were found (see Table 2). It describes the needed basic and advanced technological solutions in geriatric care that could support care giving services and aged people.

### Basic technologies for care (C2.1)

The role of simple supportive devices, like nursing beds, can help care staff to activate bed-ridden people. Also physical actions like relocation and mobilisation of clients can be done easier with available assistive aids and technologies like grab handles and patient lifts.

*‘…we have high dropout quotes. If a client is heavy and bedded low, 3 times a day turning him around, changing diaper and mobilizing is not possible. The bed is the next important thing in mobile care…’* (R7/236)

*‘…this is an interesting example…if I can transform a bed into a chair and reversed…’* (R1/77)

*‘… yes, it should simply be barrier-fee. This means beginning with the size of the grab handles, to be able to put a stool under the shower or simply a shower without barrier…’* (R6/138)

One assistive basic technology to facilitate the work of nursing staff is the adaption or adaptability of the sanitary environment. The needed technologies are helping aids in bathrooms that make the care process less stressful for the staff and could decrease the risk of falls, too. Technical solutions for relocation and movement of clients are always useful to prevent the back of the nursing staff of injuries. These kinds of helping aids are very rarely available at the customer’s homes. Furthermore, assistive devices that help elderly to stand up and mobilize, are needed.

*‘…electrically adjustable toilets and wash-bowls would influence care positively…’* (R3/48)

*‘…the whole topic “transport, transfer, embedding”. This is one thing that is bad for the back of the people, if I can say so…’* (R4/85)

*‘…another thing that would be great, the electrical mobilization aids to stand up…’* (R5/214)

Another technology that could help care givers is a door opening system for flats without an electrical door. This technology is simple and very important because often key-safes are not allowed by property management or not wanted by the clients. Also video systems to open doors remotely would be very useful for clients and care staff.

*‘…The most important thing we would need, and I think the clients too, would be the opening of the doors. It is always a dilemma to get into the flat. If someone is bed ridden… without going to the door, opening the door…’* (R6/110)

*‘…a system… for example a camera in front of the door. So she is able to see who will enter…not the key-safe … in reality it is not expensive, but it does not exist…’* (R7/535)

One interviewee explained that technical solutions for cleaning up incontinence products could be supportive for care giving staff.

*‘…if I do not need anyone who helps to clean up the incontinency material. If there would be a technology that supports in this action…’* (R1/70)

### Advanced technologies for care (C2.2)

General the experts stated that an appropriate way is needed to get informed regarding technical solutions which are already available. Therefore, the experts advised to establish an AAL-platform for information dissemination.

*‘…a platform for AAL-products should be established (the platform for helping aids of the ministry seems to be not suitable for this purpose)…’* (R3/99)

*‘…regardless if there w different providers – but accessible on one platform.’* (R8/431)

In future intelligent assistive technology will be used to foster autonomy of dependent elderly and give relatives the opportunity to have additional spare time in their lives.

*‘…spare time for relatives will be enabled to have the possibility to get out half a day without having fear. There is a huge deficit and here I see the big chances for AAL…’* (R4/42)

An often mentioned area of gadgets needed is the technology with reminder functions. For example medicine dispensers that detect, if a drug had been taken or not would be a valuable assistance. Also devices which have reminder functions for water intake do make additional sense in geriatric care.

*‘…to the automatic medicine box, when taking out the medicine it does not necessarily mean that I took the medicine. Therefore it could also trigger an alarm with the same technology…’* (R1/13)

*‘…a device which stands beside the bed like a little television and shows a glass of water; … a television…that says “Mrs. Mayer, please drink a glass of water”…’* (R7/429)

The experts explained that possibilities to interact with the devices more easily are recommended. One approach is the manipulation of the systems via speech recognition. Most experts declared that a remote control via speech recognition is strongly needed to give impaired elderly an appropriate chance of interaction with assistive devices. Because of their declining cognitive abilities, it would be useful to manipulate television, windows, jalousies, lights, and phones via speech control.

*‘…When looking at old people I recognize that there should be much more possibilities of speech recognition. When I become older I will not see the remote control (buttons) as good as now. It would be better if I could give commands to the television…’* (R1/373)

*‘…a phone … that is dialling via voice recognition. None of our clients has such a thing…’* (R7/293)

Experts also described solutions to improve social interaction. One method is the video-communication facilitated by television.

*‘…visual communication is a topic…Two models: a client has the possibility to get in contact with the organization actively via web – regardless if by television or something else. Via camera… Because the client wants to get a brain-training or a nutritional advisory service…’* (R8/257)

The next approach how technology could improve the interaction of care organisations and elderly is to have the possibility to call the service provider on demand. If advice for daily challenges is needed, the qualified staff could give it just in time to the supported person.

*‘…it would be an ideal solution if I could say, “I offer this”, as mobile care service provider. The people press a button and we visit them only when they really need us…’* (R5/139)

The experts reported that measuring activity and behaviour via monitoring systems could help the care service provider to optimize its supportive tasks based on the individual’s perceptions and habits.

*‘…I could imagine that – before providing care services – to make a technical supported activity check over 3 weeks; what does he or she do on his or her own? What is he or she not able to do? Aiming to provide optimal services to him/her. Because we are supporting in these fields where the human being is not able to act on his or her own anymore…’* (R1/156)

There is also demand for automatic light adaption, which would be useful to prevent falls.

*‘…one topic is light, when getting up at night, there are products of company XY and so on. Guidance systems that recognize activity and turn on the lights…’ (R4/95)*

The most important solutions include products which enable monitoring of vital parameters of dependent persons. Here telemedicine and telecare do play a major role for the interaction between care giving institutions, physicians and emergency services. Fall detection systems, location detection and autonomous behaviour recognition solutions with automatic interpretation were mentioned in context with telemedicine solutions. Today, available emergency-call systems can also be coupled with fall detection systems. For disoriented persons systems can be useful that are able to detect their location more easily (stand-alone-locations detectors).

*‘…fall detection systems, these things we do not have at the moment in our organization… This is a very good thing, because it gives the client a feeling of being secure…’* (R7/148)

*‘…fall detection systems are in use in our emergency call devices. With wristbands and neckbands…’* (R10/160*)*

*‘…it would be beneficial, if not only we (the care provider) would get the data. Also the physician should have access…’* (R1/331)

*‘…do you know the emergency call systems? There is a box with a voice speaking out of it. This is a very important contact person for a lot of clients. …they wake up at night and are a little bit of perplex…’* (R7/189)

*‘…a location detection system for disoriented persons who got lost…’* (R2/233)

Emergency-call systems do have a positive influence on caregiving services, because in situations where disoriented persons need support personal contact can be realized.

*‘… and these systems tell us: did he go outside? … The new systems are able to do more, e.g. it could inform: “client leaves the house”…’* (R7/63)

As mentioned it is very important for care services to gather and record vital parameters in the field of telemedicine. AAL could play a supportive and activating role by enabling people to do their daily measurements of e.g. blood pressure on their own. By enabling dependent elderly to do these necessities on their own the resources of care providers can be preserved. Nevertheless this information is not enough for providing professional care services. The data has to be supplemented with additional information about the client’s day and the personal situation while doing the measurement, e.g. an exciting talk before measuring blood pressure could increase the values.

*‘…I think telemedicine and telecare will be a topic. For support at home…’* (R1/101)

*‘…the vital parameters. For example blood pressure. This is strongly connected to the agility of the person…’* (R8/86)

*‘…the transmission of vital parameters and other data relevant to medicine or care. …this would be a benefit, if transmitted, to a call centre…*’ (R8/220)

*‘…Every person is able to measure blood pressure on his own. What is relevant for us? For us the following is relevant: Did he measure blood pressure? And additional: What happened? What did he do the whole day? So we are able to draw conclusions. The numbers and values as on its own do not represent anything…’* (R10/108)

### Health and quality of life by AAL (C3)

This category summarizes the findings how intelligent assistive technologies, smart homes, and AAL solutions can influence health and QOL of aged people.

Technologies can impact health and quality of life in elderly in various dimensions. When providing technical solutions, it is necessary to focus on and address the individual needs of elderly and also to offer products that provide additional benefits for elderly.

*‘…products… which are adapted to the need of the particular person…’* (R8/287)

*‘…by looking straight forward perhaps intelligent solutions will essentially contribute to quality and well-being and also for preservation of health…’* (R8/506)

The main objective of assistive technologies is to influence QOL of the individual by enabling elderly to live in their familiar surroundings as long as possible.

*‘…the biggest wish of all of them is to stay at home. And the longer I can stay at home by technical aids, the more my quality of life grows and I can stay at home gladly…’* (R1/188)

The experts explained that it is necessary to provide only as much technical and non-technical assistance as necessary because “over-supported” people tend to retreat themselves.

*‘…at the time he gets support, this is our experience – he tends to retreat and does nothing anymore…’* (R1/159)

*‘…it (AAL) would enable them to stay in their familiar surroundings for a long time. If a lot of things are done automatically I am able to stay at home for a longer time. That’s it…’* (R7/286)

The QOL and health can be influenced via devices which enable people to improve social contact and interaction. Technologies can have health impact by giving elderly the possibility to participate in societal actions and therefore decrease their loneliness. Another way of improving health and QOL was found in the possibility to communicate in emergency-situations directly with emergency- and care services. The health status can be positively influenced by advices from medical staff or care service providers for after-treatment questions.

*‘…I think that the intelligent devices promote health in a (health) promoting way. Especially by communicating with external contacts…’* (R1/119)

*‘…I think, for me the social inclusion is one key point … the technology enables me to live the social inclusion in that way that I can be involved more intense, then it operates like in a health promoting way. Here the technology can be supportive…’* (R6/333)

*‘…(communication) with old friends who are, for whatever reason, are not mobile, etc.…’* (R8/20)

*‘…we have to recognize AAL as a platform for social contacts. Why it is negative to communicate with the grandchild over a distance of 25.000 kilometres? I see the videoconferences. We don’t have to say ‘videoconference’; it is a personal call…’* (R10/553)

*‘… It could be hospitals, care service provider, perhaps emergency services, …’ (R8/293)*

Another way of in influencing health and QOL was found in the impact on the physical dimension. Stimulating the activity of elderly people improves the mobility of them. This aspect is important because also depression and sleep disorders can be influenced positively by physical mobilization. Some AAL solutions could be used for physical training and exercises. They could increase, or at least retain, the existing mobility.

*‘…And sophisticated and medical assured with sports science etc., programs for movement- and training in old age…’* (R8/510)

*‘…the systems that are developed will contain the stimulus to get outside. Health, well-being and also less depression, sleep disorders and so on. I think that technology can be supportive…’* (R1/132)

The experts explained that technology based reminders and warning gadgets and functions (for medicine intake, measurement of vital data, food or liquids) are able to improve physical health as well as individual wellbeing. Therefore, the compliance of medicine intake and the feeling of not being thirsty can be influenced. Reminders, warning functions and advisory functions do play an important role in prevention of diseases and other health threatening events.

*‘…but when I do not open it (the medicine dispenser) within the span between 07:00 and 08:00 a voice comes out of the television “I would like you to remind you that you didn’t take your medicine”…’* (R8/177)

*‘…the medicine dispenser because does remind you. It supports you with the prescribed medicine. This supports health. Because I keep my abilities at this level. This is why I got my medicine…’* (R7/604)

*‘…measurement of blood glucose. If it is at 300: “hello, something has to be done!” … Thereby the care staff, the relatives, the medical doctor arranges reactions…’* (R10/497)

*‘…I think of emergency situations. And of course, if I would say I have warning systems like these that tell me: “drink, take the medicine”, whatever, I could have an impact (on health)…’* (R9/525)

*‘…and if the milk is not free of lactose: “warning!”, and this is the advantage…’* (R10/671)

The elderly’s QOL can also be influenced by “technical helping hands” like robots, intelligent adaptable tables or seats which help the dependent person to stand up.

*‘…and the other would be a helping hand. This is the direction of care- and robot systems which are currently under development…’* (R6/62)

*‘…here it is the topic of handling mobility… if the table rolls away … to have support in that way, however it may be realized…’* (R7/616)

Health and QOL can also be affected by AAL solutions by compensating mental disabilities. Experts mentioned that reminder functions can have positive effects at the self-determination by reducing their daily mental stress of forgetting something important. Here, item-related reminder functions (for example: to find things/ forgetting keys) or space related reminder functions (for example: checklist when leaving flat) were described.

*‘…aids to remember in the household or in the flat. That can take away the stress a little bit: “did I do this and that, did I turn off the stove?” These things that support me by remembering and organizing the daily life…’* (R2/81)

*‘…the key that tells me “take me with you” if I leave the flat. The key wouldn’t tell me that, the system does… and applications to stimulate and help me to remember, I mean applications with touch screens for memories, pictures, music…’* (R2/234)

*‘… with RFIDs (computer chips for radio-frequency identification) that help the people to find things they lost or cannot remember the place where they left it…’ (R2/245)*

Mental health can also be improved by reducing fear, enhancing the feeling of being secure and thereby improving a more relaxed behaviour. AAL solutions can reduce fear by providing reliable emergency-systems that automatically react in the case of emergency. This is important, because people tend to be less mobile after falls which improves the physical degradation anyway. Another possibility is to enable memory trainings for people with dementia.

*‘…these reminders shall facilitate human beings to be self-determined and active. It is worthless, if a machine takes over all activities…’* (R10/206)

The mental well-being and fear can also be reduced by security-systems (in terms of automatically switching electronic devices) that are convenient for elderly because they don’t need to be worried about any switched on devices while being not at home.

*‘…the systems which provide security…’* (R9/35)

*‘…then it would be the security topic, all these things like switching off the stove…’* (R4/62)

*‘…if I forget to switch off the stove. After a defined time it switches off automatically – that’s it.’* (R6/184)

*‘…for them the aspects of being secure is not bad. I think, they feel a bit more relaxed and less anxious…’* (R6/342)

*‘…these things help because they take away the anxiety… If I would fall to the ground two times, I am afraid. When I am afraid I will fall again more easily…’* (R7/116)

One expert explained that AAL solutions could increase social and psychological wellbeing by giving elder persons duties and responsibilities.

*‘…If I would have an AAL-system, that tells me “good morning mister XY”, and furthermore shows me the activities. And then I measure the blood pressure and it tells me “thank you for your data”… this sounds strange… but sometimes it could be an essential activity. And this could be the task of AAL…’* (R10/520)

Another aspect is that self-confidence can be improved by offering AAL services which can be planned by elderly on their own and therefore make them less independent (e.g. transport services, shopping via television).

*‘…to be valued again. Imagine you do not have a duty. You are sitting around the whole day and looking into the sky. What would happen to your self-esteem? …’* (R10/531)

The experts explained that applying security solutions can have positive influential aspects to the relative’s conscience. Emergency and security solutions are often bought by elderly to calm their relatives.

*‘…yesterday, I saw a movie about residential care. And there is an old woman that says: “my daughter is calmed since I have this emergency wrist band, this is great”…’* (R9/269)

The (partially) manipulation of the home environment could enhance QOL by providing more comfort at home. The elderly’s comfort could also be enhanced by giving them the possibility to remote control/trigger (for example: via voice recognition) lights, radiators and windows. Another comfort improving technology is an automatically assistive device which enables impaired people to take a bath independently.

*‘…for me the ecologic components (radiators) of smart homes are more important…’* (R9/254)

*‘…or if I lie in my bed and lower the jalousies without standing up, this is…, let’s say, if we are in need of care, and this has a particular benefit…’* (R6/180)

The experts stated that there could be also negative impacts on QOL and health by AAL. For example the electro smog that comes along with technologies could have bad consequences for health.

*‘…in our thoughts, we want less electronic devices where we sleep. And with this (AAL), I will have it exactly there. If the old person lives in one or two rooms, then I will have it (electro smog) there…’* (R9/329)

Monitoring systems could prevent people from living a risky life on their own by being over-supervised by a technical system. This means that the right of living risky and self-determined could be undermined by AAL.

*‘…there is the aspect of controlling someone. And I, as an old person, have the right to live risky. … the surrounding environment (AAL) limits them (their liberty)…’* (R6/209)

*‘… and I don’t want to be put under tutelage by a smart home only because it wants me to drink a litre. Then I simply don’t want this. I think I can decide on my own where I want to go…’* (R9/281)

## Discussion

This study shows that AAL and assistive technologies can have beneficial impacts in several dimensions of QOL and geriatric care from the expert’s perspective. The holistic and multidimensional approach of the bio-psycho-social model of Engel [12] was the most appropriate way to get all results under one conceptual umbrella as it involves the various facets of QOL and health. This is why the results are presented by assigning them to these different dimensions (see Tables 3 and 4).

**Table 3:** **Impacts of technical solutions to QOL and health of elderly**

Technical solution	Impact on QOL and health	Dimension
Automatic switching devices (e.g. stove)	• Feeling secure	Mental
	• Feeling more relaxed	
	• Less anxiousness	
Call-on-demand systems	• Reducing retreatment	Social
emergency call systems	• Calm relatives	Mental
	• Feeling more secure	Mental
	• Directly communication with care giver	Social
	• Direct advisory of medical staff in critical situations	Physical
Fall detectors	• Reaction when threatening event occurs	Physical
Intelligent furniture/mobilization aids	• Enhanced autonomy	Physical and mental
Medicine dispensers	• Medical compliance	Physical
Memory trainings on AAL-System	• Positive impact an people	Mental
	• With declining mental capacity	
Mobility aids for self-bathing for impaired	• Enhance autonomy	Mental
Planning services (transport, shopping via television)	• Can do it on my own without help	Mental
Reminder and warning functions (medicine intake, vital data, drinks)	• Better medicinal compliance	Physical
	• Compensation of not feeling thirsty	
Reminder functions (medicine, key finders)	• Compensation of mental disabilities	Mental
Remote control of lights, windows, radiators	• Enhance comfort	Physical and mental
Task planning services	• Have duties and responsibilities (self-esteem)	Mental
Training devices/ electronically physical advisor	• Increase or retain mobility (positive impact of activity on sleep disorders)	Physical
	• Positive impact of activity on depressive diseases	Mental
Video communication systems	• Improve social interaction	Social
	• Improve care measures regarding elderly’s needs	Physical and mental
AAL-Systems (general)	• Independent living at home	Mental
	• Autonomy	
AAL-Systems (general)	• Being over-supported	Social
AAL-Systems (general)	• Electro smog	Physical and mental
AAL-Systems (general)	• Focusing on individuals needs	Mental

**Table 4:** **Impacts of technical solutions to care givers**

Technical Solution	Impact of AAL	Dimension
AAL General	• Have additional spare time for caring relatives	Physical/mental
Aids for mobilization and relocation	• Less burdening for care staff	Physical
Behaviour recognition	• Better focus on needs possible	Organizational
Electrical doors	• Easy access to client	Mental
Emergency call systems	• Reaction if threatening event	Organizational
Fall detection systems	• Reaction if threatening event	Organizational
Location detections	• Finding lost elderly very fast	Organizational
Monitoring systems for analysis of activity and behaviour	• Optimize supportive actions	Organizational
Systems for recording vital data	• Preservation of resources by enabling people	Organizational

Previous research showed that physical and mental health problems are relevant risk factors for elderly’s autonomy. Thus, it will be a relevant option to apply technologies to address needs in later life [13]. It is necessary to realize that old persons who are dependent have the same basic needs as everyone else (e.g. social interaction, housing, autonomy, control of their lives, etc.) [14].

The most important needs of elderly which were articulated are social interaction, person-centred support, continuity in life, self-determination, having fun and enjoy life. The reported problems are the decline of social involvement, the feeling of being insecure, not being needed anymore, physical, and mental limitations. From care givers’ perspective the decline of mobility, sensory abilities, and dementia diseases are the most stressing problems.

AAL solutions can enable elderly people to live a self-determined life if the systems are adjusted to their needs and seek to compensate their problems. Especially for geriatric care services, there are positive influences on the organizational level of measures. Here, the AAL technologies like fall detection, behavioural analysis systems, location detectors, and recording systems for vital data can help to react just in time when assistance or need-focused support is required. Furthermore, the resources of care givers can be preserved by optimizing their care activities based on monitoring systems. For relatives of impaired elderly people, those technologies can provide the possibility to get additional spare time, and therefore have a positive impact on mental health of them. Technical aids for mobilization could make the work of care services less burdening.

For elderly, there are several impacts on a high number of dimensions in QOL and health by applying different systems. The physical domain of health can be influenced with telemedicine and telecare services by providing advisory services for critical situations. Intelligent AAL-devices like medicine dispensers and reminder functions can affect the medicinal compliance and help to compensate mental disabilities of people. Training applications are a good vehicle to enforce elderly subjects to mobilize and train their body on their own.

Mental health can be positively influenced by enhancing autonomy and independent living by giving old people the feeling of being secure. To enable this, there are several solutions like fall detection systems, emergency call systems and automatic switching devices. Autonomy, individual comfort, and security can be impacted by applying mobilization aids like intelligent furniture or remote controlled smart home components like automatic light adaption, window control and heating equipment.

Important elements of AAL solutions are video communication systems, which enable the social inclusion and involvement of elderly people. These systems are especially important, if the dependent person lives in a rural area without frequent visits of relatives, friends or care givers.

In 2002 Van Bronswijk defined different domains of applications where technology can influence QOL [15]. Beneath supportive, preventive and compensative influences that are provided by technologies he reported that technologies for enhancement and satisfaction will become more popular. Our findings confirm this perception in that way that socio-technical, mobility- and comfort-enabling AAL solutions are impacting QOL.

Other available literature of QOL in context of AAL and other technology based support systems focuses on the quantitative evaluation of QOL and health without analysing the modes of how technologies affect these parameters [16–18]. Objective and subjective evaluations provide important information to understand QOL and health outcomes. For dement elderly especially only subjective and user-centred methods, which focus on the individual needs, lead to sufficient information about its impacts [19]. Referring to the investigative questions of this study the lack of existing literature do represent the technology-driven approach of research in the field of AAL where the influences on future users are not taken into account in a sufficient amount until today.

There were some possible negative influences of assistive technologies and AAL found. We identified that there could be a possible threatening because of electro smog in the home of elderly. Another adverse aspect is the potential of getting over-supported by intelligent devices that take over essential tasks in daily life. Due to the aim of AAL to enable a self-determined life of elderly – and therefore enhancing QOL - also ethical considerations have to be undertaken. By applying such systems, e.g. for behaviour monitoring or location detection, there must be a balance between privacy and protection according to their individual needs and abilities [5]. AAL solutions may not be understood as a tool to enhance social inclusion and safety while paying the price of getting overprotected or disciplined by others. This could lead to a lost self-determination via AAL [20].

### Strengths and limitations

In our study, experts of care giving organizations in eastern Austria were interviewed. The involved persons are working in different fields of expertise of care service providers. Because of these different professions, the results are balanced concerning the different occupational views of the interviewees. However, the interviewed persons were only from eastern Austria. In some cases this fact could make the findings only conditionally transferable to other regions and cultures. Another limiting issue is that the personal aspects of elderly (needs, problems, health and QOL) were collected only from the caregivers’ angle of view. Within this study it was not possible to gather this information from the perspective of aged people but it will be taken into account in further research activities to triangulate the results of this manuscript.

A limitation of this study may be seen in the fact that the interviews and the content analysis were performed by the same person. Strength of the study is the saturation of topics in relation to the sample size which was reached after the 8^th^ interview.

During the interviews the different understanding what AAL does mean and which technologies are subsumed under this term was a problem, This finding proves the fact that there is no national and international consensus on the terminology of these products and solutions [21].

For further understanding of the impact of AAL it will be highly important to organise interviews with aged people regarding their experiences with assistive technologies. Additionally, it is important to do interventional studies in the field of AAL to investigate potential influences, both, positive and negative, of assistive technologies and AAL solutions on health and QOL.

## Conclusion

Our findings suggest that there is a wide range of possible impacts of AAL on QOL and health of elderly and care givers. Because of the technology-focused character of research in AAL, there is a lack of resources that describe the interplay of technology and health as well as QOL from a multidimensional angle of view.

This study showed that also indulgence and person centred support do play an important role in the daily life of impaired elderly.

The results show that technology and AAL can have beneficial effects on the perceived needs of old people. The supportive role to compensate or minimize problems of aged people does represent one of the main considerable advantages of AAL.

The application of supportive technologies which enhance the feeling of being secure can lead to valuable contributions to QOL and health. Here, also quite simple technologies like electric door openers were identified.

Telemedicine and telecare technologies as well as smart home technologies seem to have the biggest potential for the future of care giving organisations. These sophisticated solutions can help to optimize their services according to their clients’ needs and problems.

Quite in contrast to these complex solutions elderly will prefer simple technologies which are technical aids (for example: grab handles), video communication and emergency call systems. Those systems are not directly connected to the meaning of or even be one part of AAL solutions.

Further research and development of new AAL solutions should focus on the investigation how needs of elderly and care givers can be addressed by these solutions by describing the pathways how AAL can influence health and QOL.

## References

[1] Krajic K, Nowak P, Rappold E (2005). Pflegenotstand in der mobilen Pflege. Diagnosen und Lösungsmöglichkeiten. Wissenschaftliches Gutachten gefördert durch die Fachgruppenvereinigung Gesundheitsberufe im ÖGB.

[2] Meyer S, Mollenkopf H (2010). AAL in der alternden Gesellschaft - Anforderungen, Akzeptanz und Perspektiven; Analyse und Planungshilfe, vol. Bd. 2. Berlin.

[3] Ambient Assisted Living Joint Programme - ICT for ageing well: *Ambient Assisted Living Joint Programme - ICT for ageing well*. [http://www.aal-europe.eu/]

[4] European Next Generation Ambient Assisted Living Innovation Alliance: *European Next Generation Ambient Assisted Living Innovation Alliance*. [http://www.aaliance2.eu/] 10.1515/bmt-2013-4246 24042898

[5] Schulke AM, Plischke H, Kohls NB (2010). Ambient Assistive Technologies (AAT): socio-technology as a powerful tool for facing the inevitable sociodemographic challenges?. Philos Ethics Humanit Med.

[6] Winkler I, Matschinger H, Angermeyer MC, Group W-O (2006). The WHOQOL-OLD; A Questionnaire for intercultural measuring of quality of life in the elderly. Psychother Psychosom Med Psychol.

[7] Bowling A (2005). Measuring health - A review of quality of life measurement scales.

[8] Naidoo J, Wills J (2009). Foundations for health promotion.

[9] Gläser J, Laudel G (2010). Experteninterviews und qualitative Inhaltsanalyse: Als Instrumente Rekonstruierender Untersuchungen.

[10] Kuckartz U, Dresing T, Rädikesr S, Stefer C (2008). Qualitative Evaluation - Der Einstieg in die Praxis.

[11] Mayring P (2010). Qualitative Inhaltsanalyse - Grundlagen und Techniken.

[12] Engel G (1977). The need for a new medical model: A challenge for biomedicine. Science.

[13] Comyn G, Olsson S, Guenzler R, Özcivelek R, Zinnbauer D, Cabrera M (2006). User needs in ICT research for independent living with a focus on health aspects.

[14] Shakespeare T (2000). The social relations of care.

[15] JEMH B v, Bouma H, Fozard JL (2002). Technology for Quality of Life: an enriched taxonomy. Gerontechnology.

[16] Cheng SC, Huang MZ, Wu LC, Chou CC, Cheng CN, Jhang SS, Shiea J (2012). Building blocks for the development of an interface for high-throughput thin layer chromatography/ambient mass spectrometric analysis: a green methodology. Anal Chem.

[17] Matlabi H, Parker SG, McKee K (2011). The contribution of home-based technology to older people’s quality of life in extra care housing. BMC Geriatr.

[18] Damant J, Knapp M, Watters S, Freddolino P, Ellis M, King D (2013). The impact of ICT services on perceptions of the quality of life of older people. J Assist Technol.

[19] Peterson CB, Prasad NR, Prasad R: **The future of assistive technologies for dementia.** *Gerontechnology* 2012.,**11**(2).

[20] Manzeschke A, Weber K, Rother E, Fangerau H (2013). Ethische Fragen im Bereich Altersgerechter Assistenzsysteme.

[21] Martin S, Kelly G, Kernohan WG, Bernadette M, Nugent C (2008). Smart home technologies for health and social care support. Cochrane Database Syst Rev.

[CR22] The pre-publication history for this paper can be accessed here: http://www.biomedcentral.com/1471-2318/14/112/prepub

